# Prevalidation of an Acute Inhalation Toxicity Test Using the EpiAirway *In Vitro* Human Airway Model

**DOI:** 10.1089/aivt.2018.0004

**Published:** 2018-06-01

**Authors:** George R. Jackson, Anna G. Maione, Mitchell Klausner, Patrick J. Hayden

**Affiliations:** MatTek Corporation, Ashland, Massachusetts.

**Keywords:** acute inhalation toxicity, EpiAirway, *in vitro* human airway model

## Abstract

***Introduction:*** Knowledge of acute inhalation toxicity potential is important for establishing safe use of chemicals and consumer products. Inhalation toxicity testing and classification procedures currently accepted within worldwide government regulatory systems rely primarily on tests conducted in animals. The goal of the current work was to develop and prevalidate a nonanimal (*in vitro*) test for determining acute inhalation toxicity using the EpiAirway™ *in vitro* human airway model as a potential alternative for currently accepted animal tests.

***Materials and Methods:*** The *in vitro* test method exposes EpiAirway tissues to test chemicals for 3 hours, followed by measurement of tissue viability as the test endpoint. Fifty-nine chemicals covering a broad range of toxicity classes, chemical structures, and physical properties were evaluated. The *in vitro* toxicity data were utilized to establish a prediction model to classify the chemicals into categories corresponding to the currently accepted Globally Harmonized System (GHS) and the Environmental Protection Agency (EPA) system.

***Results:*** The EpiAirway prediction model identified *in vivo* rat-based GHS Acute Inhalation Toxicity Category 1–2 and EPA Acute Inhalation Toxicity Category I–II chemicals with 100% sensitivity and specificity of 43.1% and 50.0%, for GHS and EPA acute inhalation toxicity systems, respectively. The sensitivity and specificity of the EpiAirway prediction model for identifying GHS specific target organ toxicity-single exposure (STOT-SE) Category 1 human toxicants were 75.0% and 56.5%, respectively. Corrosivity and electrophilic and oxidative reactivity appear to be the predominant mechanisms of toxicity for the most highly toxic chemicals.

***Conclusions:*** These results indicate that the EpiAirway test is a promising alternative to the currently accepted animal tests for acute inhalation toxicity.

## Introduction

Knowledge of acute inhalation toxicity potential is important for establishing safe handling, packaging, labeling, transport, and use procedures for chemicals, and for formulating responses to emergency exposures. Evaluation of acute inhalation toxicity potential is therefore a mandatory regulatory requirement for chemical products utilized in international commerce. Recent initiatives, such as the U.S. Environmental Protection Agency (EPA) High Production Volume (HPV) Chemical Challenge, the European Union (EU) Registration, Evaluation, Authorization and Restriction of Chemicals (REACH) program, and the Frank R. Lautenberg Chemical Safety for the 21st Century Act, have further increased the need for inhalation toxicity information for companies that produce and distribute chemicals and household consumer products on the global market.^1–4^

Specific U.S. federal agencies that have the responsibility for establishment and enforcement of hazard communication regulations, including those for inhalation toxicity of chemicals, are the Consumer Product Safety Commission (CPSC), the EPA, and the Occupational Safety and Health Administration (OSHA). OSHA regulations govern the hazard communications ubiquitously displayed on material and chemical safety data sheets (SDSs). The EPA maintains authority to require testing of chemicals under the Toxic Substances Control Act (Office of Pollution Prevention and Toxics, OPPT) and testing of pesticides under the Federal Insecticide, Fungicide, and Rodenticide Act (FIFRA; Office of Pesticide Programs, OPP). The Federal Hazardous Substances Act (FHSA) is one of the laws administered by CPSC. The European Chemicals Agency (ECHA), which requires chemical manufacturers to assess the risks posed by chemicals and provide appropriate safety information in the EU, administers the REACH regulation.

**FIG. 1.**
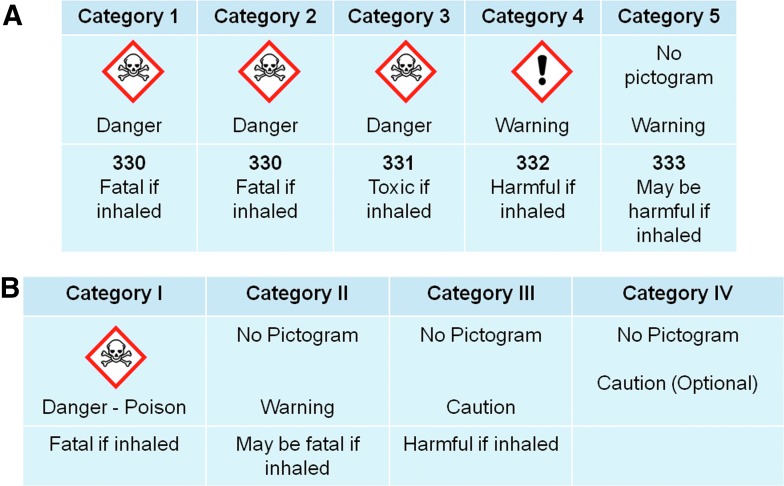
**(A)** GHS system for acute inhalation toxicity. **(B)** EPA system for acute inhalation toxicity. EPA, Environmental Protection Agency; GHS, Globally Harmonized System.

A United Nations treaty endorsed by countries, including the United States, EU member countries, China, Japan, Australia, and others, outlines a “Globally Harmonized System” (GHS) of Classification and Labeling of Chemicals.^5^ The OSHA Hazard Communication Standard is aligned with the GHS Classification and Labeling of Chemicals.^6^ The GHS specifies five acute inhalation toxicity classes with associated labels and warning phrases. If data indicate the mechanism of toxicity to be corrosivity, the substance may also be labeled as corrosive to the respiratory tract.^5^^(Section 3.1.2.6.5)^ The EPA has established a separate acute toxicity classification system that utilizes four toxicant categories for pesticides and other chemicals.^7,8^ Testing of chemicals that are expected to cause marked pain and distress due to corrosive or irritating properties is not required by the EPA system.^7,8^ The CPSC utilizes a system that includes labeling for two toxicant classes.^9^ Acute inhalation toxicity classification systems utilized by OSHA (GHS) and EPA are summarized in Figure 1A and B.

The commonly accepted test procedure for determining acute inhalation toxicity classification within the GHS and other systems requires the use of animals to conduct *in vivo* inhalation toxicity tests (OECD test guidelines [TGs] 403, 433, and 436). These TGs are based on LC50 concentrations (concentration required to cause death of 50% of the test animals; TGs 403, 436) or evident toxicity (TG 433).

**FIG. 2.**
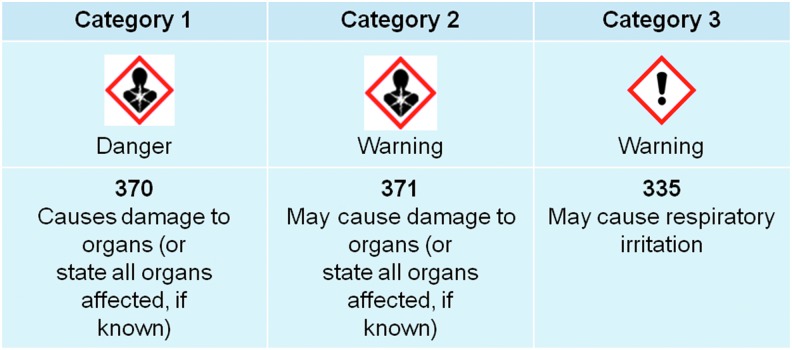
GHS system for specific target organ toxicity-single exposure.

Additional classification and labeling phrases based on significant nonlethal toxicity caused by a single inhalation exposure are also considered by the GHS under specific target organ toxicity-single exposure (STOT-SE; Fig. 2).^5,10^ STOT-SE classification is based primarily on human data (Categories 1 and 3) or animal data (Category 2). However, there are currently no standardized accepted animal test methods for STOT-SE.^5^

The high cost associated with testing all of the chemicals impacted by the REACH, the HPV Chemicals Challenge, and the Lautenberg Act (more than 30,000 existing chemicals plus all newly introduced chemicals and mixtures) poses a tremendous burden on chemical and consumer product producers.^1–4^ The extremely large number of animals needed to conduct the required inhalation toxicity testing has prompted inclusion of provisions in the REACH program that mandate utilization of alternative nonanimal tests whenever possible.^11^ The ECHA now requests alternatives' information from registrants who submit new testing proposals for vertebrate animal tests. This action is intended to ensure the avoidance of unnecessary animal testing. The National Research Council Report “Toxicity Testing in the 21st Century: A Vision and a Strategy”^12^ also recommends the replacement of animal tests with relevant *in vitro* human-based test systems. In line with these goals, U.S. government regulatory agencies consider the development and validation of alternative *in vitro* methods for acute toxicity testing, including acute inhalation toxicity testing, to be a high priority.^13^ The U.S. EPA has a stated goal of reducing *in vivo* testing for the acute toxicity “six-pack,” which includes acute inhalation testing of required tests for pesticides.^14,15^

Despite the increasing need for development and validation of an economical, standardized, and accurate *in vitro* procedure for determining acute inhalation toxicity potential, there are currently no approved nonanimal tests available for this purpose. The goal of the current work was to develop and prevalidate an *in vitro* test for determining acute inhalation toxicity as a potential alternative for currently accepted animal tests.

## Materials and Methods

### The EpiAirway model

A commercially available *in vitro* organotypic model of human mucociliary airway epithelium (EpiAirway™) was utilized for these studies (AIR-100; MatTek Corporation, Ashland, MA). The EpiAirway model is cultured at the air/liquid interface to provide a differentiated *in vivo*-like epithelial structure with barrier properties, metabolic functions, and apical dosing capabilities.^16^ Briefly, to produce the cultures, human airway epithelial cells were seeded onto microporous membrane inserts and cultured submerged in growth media until a confluent epithelial layer was attained. Cultures were subsequently raised to the air/liquid interface using differentiation promoting media to yield well-differentiated cultures exhibiting properties of native tracheobronchial tissue. These properties include a pseudostratified morphology, the presence of multiple cell types (mucus producing goblet cells, ciliated cells with actively beating cilia, basal cells, and club cells), functional tight junctions, and barrier function that can be evaluated by measurement of transepithelial electrical resistance.^16^

The EpiAirway model is produced under Good Manufacturing Practices (GMP) conditions and rigorous quality control standards to ensure long-term lot-to-lot reproducibility and reliable performance. It has been successfully utilized by numerous research laboratories worldwide in applications including toxicology, drug delivery, pharmacology, and infectious disease research.^17–28^ A complete bibliography of additional peer-reviewed research articles that include EpiAirway data is available from MatTek (www.mattek.com).

### Test chemicals

Test chemicals were selected to include a broad range of chemical structures and reactive functionalities, including acids, bases, oxidants, aldehydes, ketones, esters, alcohols, amines, halogenated aromatics, and phenol and pyridine derivatives, for which existing *in vivo* human or animal inhalation toxicity data are available. *In vivo* data were curated from supplier SDSs and the Organisation for Economic Co-operation and Development (OECD) online eChemPortal database.^29^ eChemPortal data used were primarily from the ECHA Classification & Labeling inventory and the GHS-Japan (GHS-J) database. The ECHA database comprised animal data submitted by companies and compiled but not reviewed by ECHA. GHS-J classifications are based on a weight of evidence approach that includes human STOT-SE data. The GHS-J classifications are peer reviewed by a Japanese Governmental Committee under the auspices of the Japanese National Institute of Technology and Evaluation. When discrepancies between supplier SDSs, ECHA, and GHS-J classifications were encountered, a weight of evidence approach was utilized to assign classification categories. Fifty-nine chemicals were evaluated during the current reported work. The test chemicals were obtained at the highest available purity (Sigma-Aldrich St. Louis, MO).

### MTT assay for tissue viability

The 3-(4,5-dimethylthiazol-2-yl)-2,5-diphenyltetrazolium bromide (MTT) and extractant reagents are supplied as a kit (MatTek) and were prepared following the supplier's recommendations. Tissues treated with 100 μL of 0.2% Triton X-100 were included as a positive control treatment for toxicity. EpiAirway tissues (Air-100 format) were transferred to 24-well culture plates containing 300 μL of MTT reagent per well and incubated in a 37°C, 5% CO_2_ incubator for 90 minutes. Following the incubation, the EpiAirway AIR-100 tissues were submerged in 2.0 mL of MTT extractant (MatTek). Tissues were maintained in extractant overnight. The following day, 200 μL of extractant from each tissue was transferred to a clear 96-well plate and absorbance was measured at 570 nm with background at 650 nm subtracted. The viability of test chemical-exposed tissues was calculated relative to vehicle controls using the equation: Relative Viability = [OD_sample_/Mean OD_neg ctrl_] × 100. All control and treatment groups included *n* = 3 replicates.

### Assay protocol

Development of *in vitro* inhalation toxicity screening methods that are widely applicable to numerous chemical materials, and that are amenable to high-throughput screening, is challenging to achieve due to dosimetry issues. To avoid the need to generate and characterize an aerosol, dust, mist, or gas-phase atmosphere for each chemical or material to be tested, the approach of directly applying test material solutions or suspensions to the apical surface of the EpiAirway cultures was adopted. Vehicles for preparing suspensions or solutions, including dimethylsulfoxide (DMSO), ethanol, acetone, corn oil, olive oil, and water, were evaluated. Corn oil and water were adopted as the two vehicles of choice, depending on the solubility of the test agent, because they are nontoxic to the cultures and have low volatility. Most test chemicals were soluble in one or the other of these vehicles. If a chemical was not completely soluble in either vehicle, it was applied as a suspension in either vehicle. Emulsions that separated with the test material in the top phase were not utilized. A positive displacement pipette was utilized to ensure accurate dispensing of the viscous oil solutions. To avoid evaporation of volatile test chemicals or cross exposure of tissues in adjacent wells of the culture plate during the exposure incubation, specially designed insert caps were developed and manufactured (Millicell-cm-cap, MatTek).

Before use, the EpiAirway tissues were transferred to six-well plates and equilibrated at 37°C and 5% CO_2_ with 1.0 mL of fresh EpiAirway assay medium for 18–24 hours following the supplier's recommended use protocol. The apical surface of the tissues was rinsed with phosphate buffered saline (PBS) to remove accumulated mucus, and the assay medium was exchanged with fresh medium immediately before conducting the toxicity tests.

Dose/response curves using four test chemical concentrations and the MTT viability assay were generated to determine the toxicity profile of each chemical. IC75 (the dose required to reduce the EpiAirway culture viability to 75% of vehicle-treated tissues) was utilized as an indicator of the threshold for toxicity development. This level of toxicity is also amenable to potential additional endpoint analyses involving cytokine secretion or gene expression where higher toxicity levels would be detrimental to the analysis. Applied doses were measured in units of mg/mL for convenience. Doses can be expressed as mg/cm^2^ to allow for scaling of the doses to inserts of different surface areas (inserts utilized in the current study were 0.6 cm^2^). Dose range-finding experiments using doses of 10, 50, 250, and 500 mg/mL were initially performed to determine the approximate IC75, followed by definitive experiments using doses that tightly bracketed the IC75 value as follows:

- For test chemicals with dose range-finding IC75 ≤ 10 mg/mL, definitive doses were 0.1, 0.5, 2.5, and 12.5 mg/mL.
- For range-finding IC75 between 10 and 50 mg/mL, definitive doses were 5, 15, 30, and 60 mg/mL.
- For range-finding IC75 between 50 and 250 mg/mL, definitive doses were 40, 120, 200, and 280 mg/mL.
- For dose range-finding IC75 between 250 and 500 mg/mL, definitive doses were 200, 300, 420, and 550 mg/mL.
- For dose range finding IC75 > 500 mg/mL, definitive doses were 450, 650, 850 mg/mL, and neat.

These doses were not intended to directly correlate to *in vivo* exposures, but were used to establish an empirical correlation and prediction model.

**FIG. 3.**
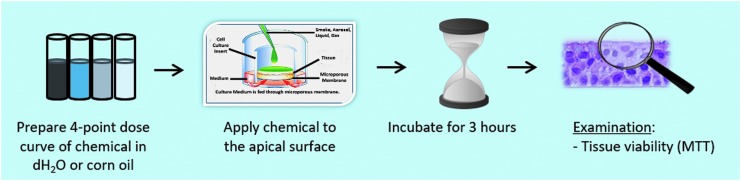
Graphical depiction of the EpiAirway™ *in vitro* acute toxicity protocol: 3-hour exposure with immediate postexposure viability determination.

The protocol is depicted graphically in Figure 3 and summarized as follows:

- Prepare test chemical at specified concentrations (mg/mL) in appropriate vehicle (water or corn oil).
- Rinse EpiAirway tissues with PBS.
- Apply 100 μL of test chemical (four concentrations of each test chemical, *n* = 3 tissues per concentration), apply insert cap.
- Incubate EpiAirway tissues @ 37°C, 5% CO_2_ for 3 hours.
- Rinse apical surface of tissues three times with PBS and immediately measure tissue viability using the MTT viability assay.

The reproducibility and interlaboratory transferability of this protocol have been previously established.^30^ The protocol has also recently been adapted for use in experiments to evaluate the respiratory irritant potential of tobacco smoke and electronic cigarette vapor.^25^

### Statistical analysis

The means and standard deviations were calculated for triplicate tissues or replicate experiments in each treatment group.

## Results

**FIG. 4.**
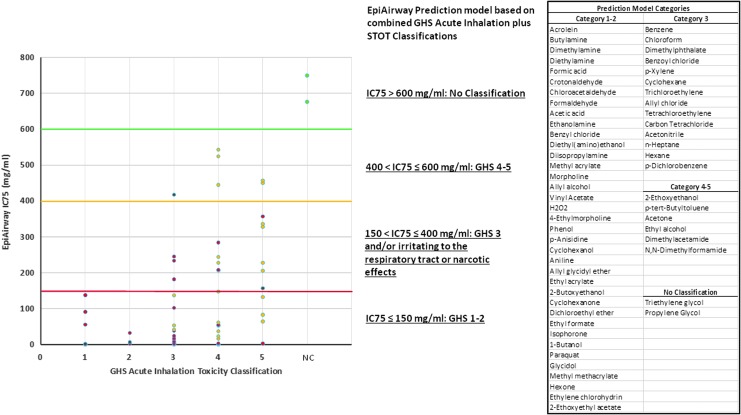
Correlation of EpiAirway IC75 toxicity values with *in vivo* determined GHS acute inhalation toxicity and STOT-single-exposure categories. Chemicals are segregated into their reported GHS acute inhalation toxicity categories (*in vivo* 4-hour rat test) along the x-axis. Chemicals with reported GHS STOT-single exposure Category 1 are shown as red dots. Chemicals with reported GHS STOT-single exposure Category 3 are shown as orange dots. Chemicals considered to be nonhazardous (no-classification) are shown as green dots. The proposed EpiAirway GHS prediction model based on a combination of GHS acute plus STOT-single exposure considerations is depicted along the y-axis. The identity of specific chemicals in each EpiAirway prediction model category is shown on the right-hand sidebar. STOT-SE, specific target organ toxicity-single exposure.

**FIG. 5.**
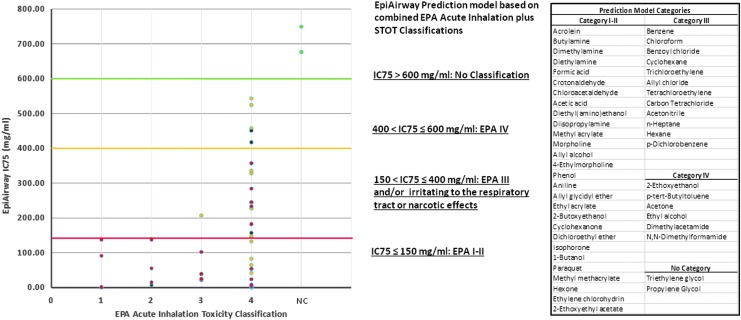
Correlation of EpiAirway IC75 toxicity values with *in vivo* determined EPA acute inhalation toxicity and STOT-single exposure categories. Chemicals are segregated into their reported EPA acute inhalation toxicity categories (*in vivo* 4-hour rat test) along the x-axis. Chemicals with reported GHS STOT-single exposure Category 1 are shown as red dots. Chemicals with reported GHS STOT-single exposure Category 3 are shown as orange dots. Chemicals considered to be nonhazardous (no-classification) are shown as green dots. The proposed EpiAirway EPA prediction model based on a combination of EPA acute plus GHS STOT-single exposure considerations is depicted along the y-axis. The identity of specific chemicals in each EpiAirway prediction model category is shown on the right-hand sidebar.

The 3-hour direct application exposure protocol with immediate postexposure viability determination was utilized to evaluate the *in vitro* toxicity of 59 test chemicals with the EpiAirway model. Comparisons of EpiAirway *in vitro* IC75 test results with GHS and EPA Acute Inhalation Toxicity classifications based on *in vivo* rat data following OECD TGs, and GHS STOT-SE classifications based on human data, are shown in Table 1. GHS dermal and ocular corrosion classifications are included in Table 1 for comparative purposes. Graphical comparisons of the EpiAirway *in vitro* IC75 values, *in vivo* rat-based GHS and EPA Acute Inhalation Toxicity classifications, and *in vivo* human-based GHS STOT-SE classifications are shown in Figures 4 and 5.

**Table 1.** EpiAirway IC75 Toxicity Responses Compared to Globally Harmonized System and Environmental Protection Agency Classifications for 59 Test Chemicals

	*Test chemical*	*Cas no.*	*GHS acute classification*^a^	*GHS STOT-SE classification ECHA/SDS*^b^	*GHS STOT-SE classification GHS-J*^c^	*EPA acute classification*^d^	*U/LRTI (SDS)*^e^	*Skin/eye corrosive*^f^	*Vehicle*	*Experimental replicates*	*Mean IC75(mg/mL)*	*Standard deviation*^g^
1	Acrolein	107-02-8	1		1(R,N,L), 3(N)	1	Y	Skin, eye	Corn oil	2	0.17	0.12
2	Butylamine	109-73-9	3		1(R,B,K)	4	Y	Skin, eye	H_2_O	1	0.71	0.05
3	Dimethylamine	124-40-3	4	3	1(R), 3(N)	4	Y	Skin, eye	H_2_O	1	0.73	0.07
4	Diethylamine	109-89-7	4	3	1,2(R)	4	Y	Skin, eye	H_2_O	1	0.75	0.11
5	Formic acid	64-18-6	3		1(B,L,K,R)	4	Y	Skin, eye	H_2_O	1	1.04	0.12
6	Crotonaldehyde	4170-30-3	1	3	3(R)	1	Y	Eye	Corn oil	2	1.81	0.43
7	Chloroacetaldehyde	107-20-0	2		1(R,O)	4	Y	Skin, eye	H_2_O	1	2.22	0.28
8	Formaldehyde	50-00-0	3		1(N,R)		Y	Skin, eye	H_2_O	1	2.98	0.29
9	Acetic acid	64-19-7	5		1,2(B,R)	4	Y	Skin, eye	H_2_O	1	3.14	0.42
10	Ethanolamine	141-43-5	4		1(N,R,L), 3(N)		Y	Skin, eye	H_2_O	1	3.27	0.25
11	Benzyl chloride	100-44-7	3	3	1(R,N)		Y	Eye	Corn oil	1	3.35	0.32
12	Diethyl(amino)ethanol	100-37-8	3		1(N), 3(R)	4	Y	Skin, eye	H_2_O	1	3.52	0.19
13	Diisopropylamine	108-18-9	2		2(R)	2	Y	Skin, eye	Corn oil	1	7.00	0.48
14	Methyl acrylate	96-33-3	3	3	1(O), 3(R)	4	Y		Corn oil	1	8.13	0.40
15	Morpholine	110-91-8	3		1(R)	4	Y	Skin, eye	H_2_O	1	8.53	0.89
16	Allyl alcohol	107-18-6	3	3	1(L,R,N), 3(R)	2	Y		H_2_O	1	14.98	1.17
17	Vinyl acetate	108-05-4	4	3	3(R,N)		Y		Corn oil	1	16.60	14.52
18	H_2_O_2_	7722-84-1	3	3	1(R)		Y	Skin, eye	H_2_O	1	17.30	1.56
19	4-Ethylmorpholine	100-74-3	4	3	2(N), 3(R)	4	Y	Skin, eye	Corn oil	1	23.63	2.90
20	Phenol	108-95-2	3		1(R,N,K,C)	3	Y	Skin, eye	Corn oil	1	24.74	1.68
21	p-Anisidine	104-94-9	2		1(B)				Acetone/O-oil	1	32.62	2.59
22	Cyclohexanol	108-93-0	4	3	3(R,N)				Corn oil	1	37.02	6.64
23	Aniline	62-53-3	3	1	1(B,R,O)	3		Eye	Corn oil	2	38.98	0.84
24	Allyl glycidyl ether	106-923	3	3	1(N,R,L,K)	4	Y	Eye	Corn oil	3	41.70	15.85
25	Ethyl acrylate	140-88-5	3	3	1(N,R), 3(N,R)	4	Y		Corn oil	1	53.48	3.28
26	2-Butoxyethanol	111-76-2	4		1(O), 3(R)	4	Y		H_2_O	1	53.61	5.12
27	Cyclohexanone	108-941	4		1(O), 2(R), 3(R)	4	Y	Eye	Corn oil	2	54.41	5.91
28	Dichloroethyl ether	111-44-4	1		1(R), 3(N)	2	Y		Corn oil	1	55.69	5.21
29	Ethyl formate	109-94-4	4	3	1(R), 3(N)		Y		Corn oil	1	61.94	11.00
30	Isophorone	78-59-1	5	3	3(R,N)	4	Y		Corn oil	3	64.59	10.14
31	1-Butanol	71-36-3	5	3	3(R,N)	4	Y	Eye	Corn oil	2	83.13	24.47
32	Paraquat	1910-42-5	1	3	1(R,K,L)	1			H_2_O	1	91.51	7.36
33	Glycidol	556-52-5	3	3	1(N,R)		Y	Eye	H_2_O	1	102.52	20.46
34	Methyl methacrylate	80-62-6	5	3	3(R,N)	4	Y		Corn oil	1	132.42	9.02
35	Hexone	108-10-1	3	3	3(R,N)	1	Y		Corn oil	1	137.31	13.13
36	Ethylene chlorohydrin	107-07-3	1		1(B,R,L,K), 3(N)	2		Eye	H_2_O	1	138.06	23.41
37	2-Ethoxyethyl acetate	111-15-9	4		3(N)	4			Corn oil	1	147.55	10.30
38	Benzene	71-43-2	5		1(N,B)	4			Corn oil	2	157.17	18.70
39	Chloroform	67-66-3	3	3	1(L,K), 3(N)	4			Corn oil	2	182.36	46.27
40	Dimethyl phthalate	131-11-3	5		3(N,R)		Y		Corn oil	1	206.36	14.84
41	Benzoyl chloride	98-88-4	4		2(R)	3	Y	Skin, eye	Corn oil	1	207.22	12.7
42	p-Xylene	1330-20-7	4	3	1(R,N,L,K), 3(N)		Y		Corn oil	1	208.06	13.54
43	Cyclohexane	110-82-7	5	3	2(C), 3(R)	4			Corn oil	1	227.96	15.36
44	Trichloroethylene	79-01-6	4		3(N,R)	4			Corn oil	1	228.03	11.77
45	Allyl chloride	107-05-1	3	3	1(R,N,K,L,C), 3(N)	4	Y		Corn oil	2	233.83	22.01
46	Tetrachloroethylene	127-18-4	4		1(N,R,L), (N,R)	4			Corn oil	1	244.08	30.8
47	Carbon tetrachloride	56-23-5	3	1	1(N,L,K)	4			Corn oil	2	245.60	8.40
48	Acetonitrile	75-05-8	4		1(N,R)	4	Y		H_2_O	1	284.72	72.1
49	n-Heptane	142-82-5	5		3(R,N)	4	Y		Corn oil	1	328.57	25.1
50	Hexane	110-54-3	5		3(N,R)	4			Corn oil	1	336.40	20.79
51	p-Dichlorobenzene	106-46-7	5		1(O), 3(R)	4			Corn oil	1	357.28	36.80
52	2-Ethoxyethanol	110-80-5	3		1(N,K,L,O)	4			H_2_O	2	417.52	51.48
53	p-tert-Butyl toluene	98-51-1	4	3	3(R,N)	3			Corn oil	3	445.13	49.05
54	Acetone	67-64-1	5	3(n)	3(R,N)	4	Y		H_2_O	1	450.69	45.61
55	Ethyl alcohol	64-17-5	5		3(R,N)	4	Y		H_2_O	2	457.57	48.61
56	Dimethyl acetamide	127-19-5	4		3(N)	4			H_2_O	1	524.84	107.07
57	N,N-Dimethylformamide	68-12-2	4		1(L), 2(R)	4			H_2_O	1	543.33	68.24
58	Triethylene glycol	112-27-6	5		NC				H_2_O	1	676.74	73.70
59	Propylene glycol	57-55-6	5		NC				H_2_O	1	750.00	60.08

^a^ GHS Acute Inhalation Toxicity classifications from Chemical SDS, or eChemPortal (ECHA or GHS-J).

^b^ STOT-SE classification from SDS or eChemPortal (ECHA).

^c^ STOT-SE classification from eChemPortal (GHS-J): B, blood; N, nervous system or narcotic; R, respiratory; L, liver; K, kidney; C, cardiovascular; O, multiple organ effects; NC, no classification.

^d^ EPA Acute Inhalation Toxicity classifications from SDS or eChemPortal.

^e^ Upper or lower respiratory tract irritation reported (SDS).

^f^ GHS acute classification for skin corrosion or serious eye damage (SDS).

^g^ Standard deviations were calculated from replicate experiments when performed, or from the replicate tissue responses (*n* = 3 per chemical) within single experiments.

ECHA, European Chemicals Agency; GHS-J, GHS-Japan; EPA, Environmental Protection Agency; GHS, Globally Harmonized System; SDSs, Safety Data Sheets; STOT-SE, specific target organ toxicity-single exposure.

Considering first the GHS (Table 1 and Fig. 4), the data show that all chemicals with GHS Acute Inhalation Toxicity Categories 1–2 had *in vitro* EpiAirway IC75 toxicity values <150 mg/mL. The majority of the remaining chemicals with GHS Acute Inhalation Toxicity Categories ≤3 or human STOT-SE Categories 1–3 had *in vitro* EpiAirway IC75 toxicity values <400 mg/mL (Fig. 4). Therefore, prediction model cutoff limits were assigned as follows: GHS Categories 1–2 ≤ 150 mg/mL; 150 mg/mL <GHS Category 3 ≤ 400 mg/mL; and 400 mg/mL <GHS Categories 4–5 ≤ 600 mg/mL. Chemicals that are considered to be nonhazardous had IC75 values >600 mg/mL (Supplier's SDSs). Specific chemicals that fall within each of these categories are listed within the figure.

For comparison to the EPA system, prediction model cutoff limits were assigned as follows (Fig. 5): EPA Categories I–II ≤150 mg/mL; 150 mg/mL <EPA Category III ≤400 mg/mL; and 400 mg/mL <EPA Category IV ≤600 mg/mL. Chemicals with IC75 values >600 mg/mL are considered to be nonhazardous.

Using the prediction model cutoff limits selected for Figures 4 and 5, the EpiAirway IC75 results identify *in vivo* rat-based GHS Acute Inhalation Toxicity Category 1–2 and EPA Acute Inhalation Toxicity Category I–II chemicals with 100% sensitivity, but overpredict categories for many chemicals compared with the *in vivo* rat-based acute inhalation toxicity test. The specificity of the EpiAirway IC75 results compared with the *in vivo* rat-based GHS 1–2 and EPA I–II Acute Inhalation Toxicity Category chemicals was 43.1% and 50.0%, respectively. However, the chemicals that were overpredicted in comparison to the *in vivo* rat acute inhalation toxicity test were quite often classified as *in vivo* human GHS STOT-SE Category 1 toxicants. Thus, these chemicals are known to cause serious, lasting toxic effects in humans following a single exposure. The EpiAirway IC75 ≤ 150 mg/mL classification showed a sensitivity of 75.0% and specificity of 56.5% with respect to *in vivo* human GHS STOT-SE Category 1 chemicals. GHS skin and eye corrosion classifications corroborate the highly toxic nature of chemicals identified as such by the EpiAirway test (Table 1). Chemicals with a demonstrated history of low/no human acute inhalation toxicity potential, such as acetone, had EpiAirway IC75 values above 400 mg/mL, and nonhazardous vehicles such as those utilized in e-cigarette liquids (triethylene glycol, propylene glycol) were above 600 mg/mL. Contingency tables comparing EpiAirway IC75 test results with *in vivo* rat results (GHS and EPA systems) and human GHS STOT-SE categories are shown in Tables 2–4.

**Table 2.** Cross Tabulation of EpiAirway IC75 Categories Compared with Globally Harmonized System Acute Inhalation Toxicity Categories

	*GHS Acute Inhalation Toxicity Category*		
*EpiAirway IC75*	*1–2*	*≥3*	*Total*
≤150 mg/mL	8	29	37
>150 mg/mL	0	22	22
Total	8	51	58

Sensitivity: 8/8 = 100.0%. Specificity: 22/51 = 43.14%. Positive predictive value: 8/37 = 21.6%. Negative predictive value: 22/22 = 100.0%. Overall accuracy: 30/59 = 50.8%.

**Table 3.** Cross Tabulation of EpiAirway IC75 Categories Compared with Environmental Protection Agency Acute Inhalation Toxicity Categories

	*EPA Acute Inhalation Toxicity Category*		
*EpiAirway IC75*	*I–II*	*≥III*	*Total*
≤150 mg/mL	8	20	28
>150 mg/mL	0	20	20
Total	8	40	48

Sensitivity: 8/8 = 100.0%. Specificity: 20/40 = 50.0%. Positive predictive value: 8/28 = 28.5%. Negative predictive value: 20/20 = 100.0%. Overall accuracy: 28/48 = 58.3%.

**Table 4.** Cross Tabulation of EpiAirway IC75 Categories Compared with Globally Harmonized System-Specific Target Organ-Single Exposure Categories

	*GHS STOT-SE category*		
*EpiAirway IC75*	*1*	*>1*	*Total*
≤150 mg/mL	27	10	37
>150 mg/mL	9	13	22
Total	36	23	59

Sensitivity: 27/36 = 75.0%. Specificity: 13/23 = 56.5%. Positive predictive value: 27/37 = 73.0%. Negative predictive value: 13/22 = 59.1%. Overall accuracy: 40/59 = 67.8%.

For the most toxic chemicals tested in the current study, a common mechanism of action appears to be related to corrosive properties. Seventeen of the 20 most toxic chemicals identified in the EpiAirway assay are also corrosive to skin or ocular tissues (Table 1). Acidic or basic chemical properties underlie the corrosive nature for some of these chemicals (e.g., acetic acid, formic acid, butyl amine, dimethylamine, diethylamine, and ethanolamine). These chemicals tended to be GHS STOT-SE Category 1 based on human data, but Acute Inhalation Toxicity Category 3–4 (GHS) or IV (EPA) based on *in vivo* rat tests. Five of the eight Acute Inhalation Toxicity GHS Category 1–2, and 4 out of 8 EPA I–II chemicals are corrosive to skin or ocular tissues. However, oxidant (paraquat) or electrophilic reactivity (e.g., acrolein, crotonaldehyde, chloroacetaldehyde, and formaldehyde) appears to be predominant chemical features of these chemicals. Numerous nonpolar organic compounds that were classified as Acute Inhalation Toxicity Categories 4–5 (GHS) or IV (EPA) were reported to have *in vivo* human respiratory irritant or narcotic effects (GHS STOT-SE Categories 1–3; nonpolar hydrocarbons).

Among the test chemicals evaluated in the current study, GHS STOT-SE effects most often cited were respiratory effects, or combined respiratory effects with other organ toxicities (most often narcotic effect; Table 1, eChemPortal, GHS-J). There were only few reports of other organ (blood, liver, kidney, and nerves) effects without concurrent respiratory effects. Thus, the respiratory tissue appears to function as a reasonably good surrogate for identifying nonrespiratory toxic effects.

## Discussion

The work presented in the current report is a continuation of efforts toward development and validation of *in vitro* alternative models for acute inhalation toxicity testing. Willoughby and McKim^26,27^ previously evaluated a multi-endpoint approach in the EpiAirway model with a limited set of six chemicals. Willoughby found promising results for identifying respiratory toxicants using a combination of endpoints, including tissue viability (MTT assay), oxidative stress (cellular glutathione [GSH] levels), and gene expression of markers for inflammation, metabolic activity, and apoptosis. A previous study conducted by researchers at BASF (Sauer et al.^30^) tested a set of 19, mostly water-soluble chemicals in the EpiAirway model. The BASF study utilized the same 3-hour direct application protocol developed by MatTek and utilized in the current work. IC50 values based on the MTT viability assay were compared with rat *in vivo* 4-hour LC50 values classified according to EPA and GHS hazard categories. The data were evaluated for ability to distinguish toxic (GHS/EPA Categories 1–3/I–III) from nontoxic (GHS/EPA Categories 4–5/IV) chemicals. Sensitivities of 87.5%–100% and specificities of 56%–89% were obtained. However, only a modest ability to distinguish between toxicity subcategories was reported. BASF researchers suggested further testing with an expanded set of test chemicals and also emphasized the importance of determining mode of action for the observed toxic effects. The BASF researchers also noted that the EpiAirway system was highly reproducible with respect to positive and negative control treatments, and repeat evaluations of test chemicals.^30^

The current work reports results from testing of the EpiAirway 3-hour direct application protocol with a broad range of 59 chemicals, the largest set of test chemicals to date. The current work also included more water-insoluble test chemicals and introduced the use of the EpiAirway insert caps. Prediction model cutoffs in the current work were applied to distinguish four categories corresponding to the following GSH and EPA categories: highly toxic (GHS/EPA Acute Inhalation Toxicity Categories 1–2/I–II or STOT-SE Category 1); moderately toxic (GHS/EPA Acute Inhalation Toxicity Categories 3/III or STOT-SE Category 3); mildly toxic (GHS/EPA Acute Inhalation Toxicity Categories 4–5/IV or STOT-SE Category 3); and nontoxic or nonhazardous.

The data reported here build on prior reports^24–28,30^ to further demonstrate that the EpiAirway toxicity test is highly sensitive for identifying dangerous toxic chemicals and/or respiratory tract corrosives. GHS STOT-SE classifications that are based on human data confirm the toxic or corrosive nature of many chemicals that are not classified as such by the currently approved *in vivo* acute 4-hour rat test. In this regard, the proposed EpiAirway inhalation toxicity test appears to offer an additional margin of safety for protecting human health. However, the ability to define highly toxic chemicals (GHS/EPA Categories 1–2/I–II) with good specificity (i.e., low incidence of false positive responses) is important because classification to these categories has implications regarding regulatory requirements for respirator use, packaging, and shipping. Furthermore, the ability of the EpiAirway test to identify highly toxic chemicals that may specifically target nonrespiratory organs (e.g., nerve agents) will require further evaluation.

Evaluation of a material for respiratory corrosive potential is a sensible first step in assessment of potential acute inhalation toxicity, because by EPA guidance, corrosive materials should be excluded from *in vivo* inhalation testing.^7,8^ Confirmation of suspected corrosive properties following a positive EpiAirway test result may be achieved using regulatory approved *in vitro* methods for determining skin corrosion (OECD TG #431). Based on the limited chemical set evaluated in the current work, while materials that are corrosive by virtue of acidic or alkaline properties are reliably classified as human STOT-SE Category 1 materials, they do not appear to be highly lethal in the *in vivo* rat LD50 test. However, corrosive materials that have electrophilic or oxidative properties may have more lethal effects in the rat model. Confirmation of strong electrophilic or oxidative properties utilizing the newly developed EpiAirway Nrf2 reporter model,^28^ measurement of GSH levels or other oxidative stress assays,^26^ and determination of acidic or alkaline properties may prove useful for assigning a classification of GHS/EPA Acute Inhalation Toxicity Categories 1–2/I–II.

The current work was conducted using direct application of test materials in solutions to facilitate moderately high-throughput screening. However, testing of materials such as gases or aerosols, while technically more challenging and subject to lower throughput, is also possible with the EpiAirway model. Future work with aerosolized materials that more closely mimic actual exposure conditions may lead to improved correlation to *in vivo* results.

## Conclusions

In conclusion, the EpiAirway 3-hour direct application protocol appears to be a promising approach for *in vitro* determination of acute inhalation toxicity testing. A limitation of the current work is the relatively low number of GHS/EPA Category 1–2/I–II chemicals, as well as the low number of weakly toxic or nonhazardous chemicals that were evaluated. Ongoing work will evaluate an additional 100 chemicals to refine the prediction model and fill in gaps in the chemical applicability domain. Future work will also further evaluate the utility of corrosion, electrophilic stress, and oxidative stress assays for allowing subclassification of GHS/EPA Categories 1–2/I–II and Category 3/III chemicals.

## References

[1] Arts JH, Muijser H, Jonker D, et al. Inhalation toxicity studies: OECD guidelines in relation to REACH and scientific developments. Exp Toxicol Pathol 2008:60;125–133 18455380 10.1016/j.etp.2008.01.011

[2] Costa DL Alternative test methods in inhalation toxicology: challenges and opportunities. Exp Toxicol Pathol 2008:60;105–109 18486462 10.1016/j.etp.2008.01.001

[3] Bakand S, Winder C, Khalil C, et al. Toxicity assessment of industrial chemicals and airborne contaminants: transition from in vivo to in vitro test methods: a review. Inhal Toxicol 2005:17;775–787 16195213 10.1080/08958370500225240

[4] www.epa.gov/sites/production/files/2016-06/documents/tsca21_clean_integrated_v2.pdf (Accessed 515, 2018)

[5] Globally Harmonized System of Classification and Labeling of Chemicals (GHS) Sixth revised edition, 2015 www.unece.org/fileadmin/DAM/trans/danger/publi/ghs/ghs_rev06/English/ST-SG-AC10-30-Rev6e.pdf (Accessed 515, 2018)

[6] www.osha.gov/dsg/hazcom/index.html (Accessed 515, 2018)

[7] Health Effects Test Guidelines: OPPTS 870.1000 Acute Toxicity Testing—background. United States Environmental Protection Agency, Office of Prevention, Pesticides and Toxic Substances, 2002 https://ntp.niehs.nih.gov/iccvam/suppdocs/feddocs/epa/epa_870r_1000.pdf (Accessed 515, 2018)

[8] Health Effects Test Guidelines: OPPTS 870.1300 Acute Inhalation Toxicity. U.S. Environmental Protection Agency, Office of Prevention, Pesticides and Toxic Substances, 1998 www.epa.gov/test-guidelines-pesticides-and-toxic-substances/series-870-health-effects-test-guidelines (Accessed 515, 2018)

[9] 16 CFR § 1500.3. https://ntp.niehs.nih.gov/iccvam/suppdocs/feddocs/cpsc/cpsc_1500_3.pdf (Accessed 515, 2018)

[10] Hazard Communication Information Sheet reflecting the US OSHA Implementation of the *Globally Harmonized System of Classification and Labelling of Chemicals (GHS)*. www.schc.org/assets/docs/ghs_info_sheets/specific_target_organ_toxicity-single_exposure.pdf (Accessed 515, 2018)

[11] TAPIR—Appendix 12. Report of endpoint working group 4 “Irritation/Corrosion” REACH implementation project 3.3-1. Final report, 6th June 2005, 42p

[12] National Research Council (NRC). (2007). Toxicity Testing in the 21st Century: A Vision and a Strategy. Washington, DC: National Academy Press www.nas.edu (Accessed 515, 2018)

[13] Clippinger AJ, Allen D, Jarabek AM, et al. Alternative approaches for acute inhalation toxicity testing to address global regulatory and non-regulatory data requirements: an international workshop report. Toxicol In Vitro 2017:48;53–70 29277654 10.1016/j.tiv.2017.12.011 PMC8215693

[14] Process for evaluating & implementing alternative approaches to traditional in vivo acute toxicity for FIFRA regulatory use. 2016 https://www.epa.gov/sites/production/files/2016-03/documents/final_alternative_test_method_guidance_2-4-16.pdf (Accessed 515, 2018)

[15] Letter to Stakeholders on EPA Office of Pesticide Programs's Goal to Reduce Animal Testing from Jack E. Housenger, Director Office of Pesticide Programs. Mar 16, 2016 www.regulations.gov/document?D=EPA-HQ-OPP-2016-0093-0003 (Accessed 515, 2018)

[16] Bérubé K, Pitt A, Hayden P, et al. Filter-well technology for advanced three-dimensional cell culture: perspectives for respiratory research. Altern Lab Anim 2010:38 Suppl 1;49–65 21275484 10.1177/026119291003801S04

[17] Agu RU, Valiveti S, Paudel KS, et al. Permeation of Win 55, 212–2, a potent cannabinoid receptor agonist, across human tracheo-bronchial tissue (EpiAirway^™^). J Pharm Pharmacol 2006:58;1–7 10.1211/jpp.58.11.0006 17132208

[18] Leonard AK, Sileno AP, MacEvilly C, et al. Development of a novel high-concentration galantamine formulation suitable for intranasal delivery. J Pharma Sci 2005:94;1736–1746 10.1002/jps.20389 15986464

[19] Leonard AK, Sileno AP, Brandt GC, et al. *In vitro* formulation optimization of intranasal galantamine leading to enhanced bioavailability and reduced emetic response *in vivo*. Int J Pharm 2007:335;138–146 17174048 10.1016/j.ijpharm.2006.11.013

[20] Moscona A, Porotto M, Palmer S, et al. A recombinant sialidase fusion protein effectively inhibits human parainfluenza viral infection in vitro and in vivo. J Infect Dis 2010:202;234–241 20533871 10.1086/653621 PMC2891144

[21] Porotto M, Rockx B, Yokoyama CC, et al. Inhibition of nipah virus infection in vivo; targeting and early stage of paramyxovirus fusion activation during viral entry. PLoS Pathog 2010:6;e1001168 21060819 10.1371/journal.ppat.1001168 PMC2965769

[22] Freishtat RJ, Watson AM, Benton AS, et al. Asthmatic airway epithelium is intrinsically inflammatory and mitotically dyssynchronous. Am J Respir Cell Mol Biol 2011;44:863–869 20705942 10.1165/rcmb.2010-0029OC PMC3135846

[23] Bai J, Smock SL, Jackson GR Jr, et al. Phenotypic responses of differentiated asthmatic human airway epithelial cultures to rhinovirus. PLoS One 2015:10;e0118286 25706956 10.1371/journal.pone.0118286 PMC4338293

[24] Balharry D, Sexton K, BeruBe KA An *in vitro* approach to assess the toxicity of inhaled tobacco smoke components: nicotine, cadmium, formaldehyde and urethane. Toxicology 2008:244;66–76 18082304 10.1016/j.tox.2007.11.001

[25] Neilson L, Mankus C, Thorne D, et al. Development of an in vitro cytotoxicity model for aerosol exposure using 3D reconstructed human airway tissue; application for assessment of e-cigarette aerosol. Toxicol In Vitro 2015:29;1952–1962 26176715 10.1016/j.tiv.2015.05.018

[26] Willoughby JA, McKim JM Identifying respiratory Toxicity Using the EpiAirway (TM) human 3-D model combined with multiple endpoint analysis. In Vitro Cell Dev Biol Anim 2009:45:S56

[27] Willoughby JA Predicting respiratory toxicity using a human 3D airway (EpiAirway) model combined with multiple parametric analysis. Appl In Vitro Toxicol 2015:1;55–65

[28] Fields W, Maione A, Keyser B, et al. Characterization and application of the VITROCELL VC1 smoke exposure system and 3D EpiAirway models for toxicological and e-Cigarette evaluations. Appl In Vitro Toxicol 2017:3;68–83

[29] eChemPortal: Global Portal to Information on Chemical Substances. www.echemportal.org/echemportal/substancesearch/page.action?pageID=134 (Accessed 515, 2018)

[30] Sauer UG, Vogel S, Hess A, et al. In vivo-in vitro comparison of acute respiratory tract toxicity using human 3D airway epithelial models and human A549 and murine 3T3 monolayer cell systems. Toxicol In Vitro 2013:27;174–190 23085368 10.1016/j.tiv.2012.10.007

